# Optimizing diagnostic tests for persulphate-induced respiratory diseases

**DOI:** 10.1186/s13601-016-0118-7

**Published:** 2016-07-21

**Authors:** M. H. Foss-Skiftesvik, L. Winther, H. F. Mosbech, P. S. Skov, M. S. Opstrup, H. Søsted, C. Zachariae, J. D. Johansen, C. R. Johnsen

**Affiliations:** Department of Dermato-Allergology, National Allergy Research Centre, Copenhagen University Hospital Gentofte, Kildegårdsvej 28, 2900 Hellerup, Denmark; Department of Dermato-Allergology, Research Centre for Hairdressers and Beauticians, Copenhagen University Hospital Gentofte, Hellerup, Denmark; Allergy Clinic, Department of Dermato-Allergology, Copenhagen University Hospital Gentofte, Hellerup, Denmark; Department of Dermato-Allergology, Copenhagen University Hospital Gentofte, Hellerup, Denmark; Reflab ApS, Copenhagen, Denmark

**Keywords:** Specific inhalation challenge, Persulphates, Persulphate salts, Histamine release test, Skin prick tests, Occupational asthma, Occupational rhinitis

## Abstract

**Background:**

Persulphates from hair bleaching products are considered the major cause of occupational-rhinitis and asthma in hairdressers. The specific inhalation challenge (SIC) is considered ‘reference standard’ for diagnosing persulphate-induced asthma and rhinitis; however, the currently validated method of performing SIC with persulphate powder is time consuming with a duration of up to 4 days. The value of skin prick tests (SPTs) and histamine release tests (HRTs) with persulphates is unknown. The aim of this study was to establish a novel rapid SIC with persulphate powder to test for both rhinitis and asthma simultaneously in 1 day. In addition, we assessed the suitability of SPTs and HRTs for detecting persulphate-induced respiratory diseases.

**Methods:**

The study population included 19 hairdressers with a history of work-related rhinitis and/or asthma symptoms, 12 symptomatic controls (10 with concurrent allergic asthma and rhinitis and two with non-allergic asthma), and 40 healthy controls. A previous severe asthmatic reaction and/or anaphylactic reaction to persulphates was considered an exclusion criterion for hairdressers. The 19 hairdressers and 12 symptomatic controls had SIC performed with 3 × 5 min exposures to potassium persulphate powder in a provocation chamber. All participants, including the 40 healthy controls, were subjected also to SPTs and HRTs with three persulphate salts at concentrations of 2–20 % and 0.03–1 %, respectively.

**Results:**

None of the symptomatic controls had a nasal or bronchial response to SIC with potassium persulphate. Six hairdressers presented a nasal and two a bronchial response. No severe reactions occurred. No positive SPTs were recorded, neither among hairdressers, symptomatic controls, nor healthy controls. All three groups showed nonspecific non-IgE mediated histamine release to persulphates in HRT.

**Conclusions:**

The proposed method for performing SIC showed a high specificity for detecting persulphate-induced asthma and rhinitis. The rapid SIC was able to produce positive nasal and bronchial responses in symptomatic hairdressers without any severe reactions occurring. SPTs and HRTs cannot predict asthma or rhinitis caused by persulphates.

## Background

Persulphates are low-molecular weight chemicals (<10 kDA) with strong oxidizing properties and wide application in hair bleaching products. They are also found in dental prosthesis cleaners, food starch, paper and cellophane, as a reducing agent in photography, and as etching solution for printed circuit boards [1]. Persulphates can induce immediate and delayed reactions, such as contact dermatitis, contact urticaria, asthma, rhinitis, and anaphylaxis [2–6]. Reported cases of immediate type reaction caused by persulphates are predominantly among hairdressers, but also workers producing persulphates [7, 8] and consumers of hair bleaching products [3, 9] have been reported to react.

As with most low-molecular weight agents, the mechanism by which persulphates induce immediate reactions is not fully understood. Immunoglobulin E (IgE) [4, 8, 10], T-cells [11, 12], and oxidative events have been proposed to contribute to the development of persulphate-induced asthma and rhinitis [13].

When assessing a patient with possible persulphate-induced rhinitis or asthma, various tests can be considered. Several studies describe the use of skin prick tests (SPTs) [10, 14, 15]; however, validation and standardization are lacking. Only one study addressed the use of histamine release test (HRT) [16] and results were inconclusive.

The specific inhalation challenge (SIC) is held as ‘reference standard’ for diagnosing occupational-rhinitis and asthma [17]. SIC with persulphate has been performed with a realistic approach attempting to reproduce conditions in the hairdressing salon [18–20]. Typically, mixtures of persulphate powder and lactose powder [20], or bleaching powder and hydrogen peroxide [21] are tipped from one tray to another inside a specially designed provocation chamber. The test has also been performed by administering an aqueous persulphate solution with a nebulizer and by spraying the solution directly into the nose when examining asthma [22, 23] and rhinitis [12], respectively. The SIC performed with persulphate in the realistic approach has previously been validated [20]. In this validated approach, the patient is exposed to a mixture of persulphate powder and lactose powder. The exposure is performed step-wise with increasing doses of persulphate during four consecutive days. The maximal exposure on the fourth day is 30 g of potassium persulphate for 10 min. A sensitivity of 100 % and a specificity of 87.5 % for diagnosing persulphate-induced asthma were reported. A disadvantage of this approach is, that it is very time consuming for both investigator and patient.

The aim of our study was, with a focus on Munoz’ validated method, to establish a new realistic approach rapid SIC performed with potassium persulphate to test for both rhinitis and asthma simultaneously in 1 day. Instead of using the step-wise approach over several days, we exposed the patients to 30 g of potassium persulphate on the first day for 3 × 5 min. Instead of the typical tipping method, we used a new stirring method in order to obtain a more reproducible exposure. In addition, we assessed the potential for diagnosing persulphate-induced asthma and rhinitis by SPTs and HRTs using three different persulphates (ammonium persulphate, potassium persulphate and sodium persulphate) in concentrations from 2–20 and 0.03–1 %, respectively.

## Methods

The study was performed as a clinical single-blinded case–control study between February 2014 and May 2016.

### Hairdressers

Hairdressers with work-related respiratory symptoms who had either contacted the hot-line of the Research Center for Hairdressers and Beauticians or were refereed to our unit for suspected occupational asthma and/or rhinitis were eligible for inclusion in this study. Hairdressers with a history of severe asthmatic reactions and/or anaphylactic reactions to hair bleaching products were excluded. Standardized interviews were employed to obtain a detailed medical and occupational history, as well as records of atopic diseases and smoking. Respiratory symptoms suggestive of asthma and rhinitis were assessed and their association with exposure to persulphates and other hairdressing chemicals was explored. A positive stop/resume test was defined as respiratory symptoms improving after periods away from work and worsening at the workplace [24]. A physical examination that included rhinoscopy was performed to exclude nasal conditions mimicking rhinitis.

### Symptomatic controls

Individuals with a history of asthma and rhinitis without known sensitization or exposure to persulphates were recruited among patients in our unit and through an advertisement on a website for research subjects.

### Healthy controls

For the SPT and HRT with persulphates we recruited a group of healthy controls without known asthma, rhinitis, or urticaria.

Prior to any clinical tests, inhaled corticosteroids were discontinued for 2 weeks, oral antihistamine and nasal corticosteroids for 72 h, long-acting beta_2_-agonist and leukotriene receptor antagonists for 48 h, and short-acting beta_2_-agonist for 8 h. The following were considered exclusion criteria: unstable asthma during the last 3 months before inclusion, regular use of oral corticosteroids, baseline forced expiratory volume in 1 s (FEV_1_) ≤70 % of predicted normal value, recent (<4 weeks) respiratory tract infection, chronic obstructive pulmonary disease, severe hypertension, immunological diseases, pregnancy or unstable cardiovascular diseases.

### Immunologic tests

SPTs were performed in duplicate with 10 common aeroallergens (Soluprick SQ^®^; ALK-Abelló, Hørsholm, Denmark), latex, and chlorhexidine digluconate (5 mg/mL). Negative (diluent) and positive (histamine 10 mg/mL) controls were also included. A positive reaction was defined by a wheal with a diameter ≥3 mm. The SPT was only considered to be valid when the positive control was positive and the negative control was negative. Atopy was defined as a positive SPT reaction to one or more of the common allergens.

In addition, SPTs were performed with freshly prepared solutions of ammonium persulphate (ACS reagent ≥98.0 %, CAS 7727-54.0), potassium persulphate (ACS reagent, ≥99.0 %, CAS 7727-21-1), and sodium persulphate (purum p.a., ≥99.0 %, CAS 7775-27-1); all Sigma-Aldrich, St. Louis, MO, USA. The persulphates were dissolved in physiologic saline solution. Ammonium and sodium persulphate were prepared at 2, 5, 7.5, 10 and 20 (wt/vol). Potassium persulphate was used at 2, 5, and 7.5 (wt/vol), as it was insoluble at higher concentrations. The solutions’ pH ranged from 1.45 to 5. First, the lowest three concentrations of the persulphates solutions were applied. If no reaction occurred within 15 min, 10 % solution was applied. Finally, if no reaction occurred again, the test was performed with the 20 % solution. Reactions were recorded after 15 and 30 min.

Heparinized blood (5 mL) for HRT was collected at and sent to RefLab ApS (Copenhagen, Denmark) according to standard procedures. Blood samples were stored at room temperature for a maximum of 6 h prior to analysis. Persulphate solutions were prepared daily and tested at concentrations of 0.03, 0.06, 0.125, 0.25, 0.5 and 1.0 (wt/vol) in duplicates. Briefly, 25 µL aliquots were incubated with 25 µL persulphate dilutions at 37 °C for 1 h. During incubation, the released histamine bound to a glass fiber coated microtitre plate and was detected fluorometrically after coupling to *o*-phthaldialdehyde [25]. Positive reactions were categorized according to the lowest concentration producing significant histamine release (10 ng histamine/mL blood). If no histamine was released, the result was categorized as negative.

Finally, whole blood was collected, serum was separated and stored at −20 °C until total IgE was measured by the ImmunoCap^®^ assay (Thermo Fisher Scientific, Waltham, MA, USA).

### Lung function tests

Hairdressers and symptomatic controls had relevant asthma medication discontinued prior to the performance of any lung function tests. Spirometry, including reversibility test and methacholine challenge was performed for each hairdresser and control 2–3 days before SIC.

Forced expiratory flow in the first second (FEV_1_) and forced vital capacity (FVC) within 2 standard deviations (SD) of predicted normal values were considered normal. The reversibility test was deemed positive if FEV_1_ increased by ≥12 % or >200 ml upon inhalation of β_2_-agonist. Bronchial hyperresponsiveness (BHR) was assessed by the bronchial provocation test with methacholine. The provocative dose of methacholine producing a 20 % fall in FEV_1_ (PD20) was expressed in micrograms.

After spirometry, fractional exhaled nitric oxide (FeNO) was measured with a DENOX 88 analyzer (ECO MEDICS AG, Duernten Switzerland) and was considered elevated at ≥25 ppb [26].

### SIC with persulphate

SIC was performed on an outpatient basis. On a separate control day, SIC was performed with 50 g d-lactose monohydrate (Sigma-Aldrich). In the absence of a bronchial- and nasal response during the following 24 h, subjects were exposed to a mixture of 30 g potassium persulphate and 20 g lactose powder. The participants, but not the investigator, were blind to the nature of the challenges.

During exposure, participant sat at a table inside a provocation chamber (2.1 m × 2.2 m × 2.3 m) at ambient temperature and humidity. Fresh air was supplied at 0.5/h through a high efficiency particulate air and carbon filters. Test substances were contained in a 1-L Erlenmeyer flask (Schott, Mainz, Germany), placed 30 cm from the subjects’ face on a magnetic stirrer (IKAMAG^®^ RCT basic; IKA, Staufen, Germany), and swirled in the air by stirring the magnet (length: 7 cm) at 810 rpm. Maximal exposure consisted of 3 × 5 min, with 20-min intervals in between. During pauses and after maximal exposure was reached, participants were removed from the provocation chamber. Exposure was discontinued if the patient developed a significant bronchial response before maximal exposure was reached. Monitoring for a bronchial and nasal response was performed at baseline; in between each exposure; 15, 30, and 60 min after exposure; and hourly thereafter until sleep. Participants were monitored in the hospital during the first 8 h; thereafter, they performed self-measurements of FEV_1_ and nasal symptoms at home until sleep and again the following morning when waking up.

### Quantification of potassium persulphate during SIC

To assess the reproducibility of the stirring method, the amount of potassium persulphate in the provocation chamber was quantified during three challenges on three separate days. Particles sized 10–300 nm and 0.1–10 µm were counted using a NanoTracer PNT800 (Philips Electronics, Eindhoven, The Netherlands) and a Dust Trak™ Aerosol Monitor Model 8520 (TSI, Shoreview, MN, USA), respectively, placed 30 cm away from the Erlenmeyer flask.

### Evaluation of bronchial response

Airway obstruction was assessed by FEV_1_ using a portable asthma monitor (AM1; Jaeger, Hoechberg, Germany). A sustained ≥15 % decrease in FEV_1_ from baseline was considered a positive result for asthma, provided that fluctuations in FEV_1_ were ≤10 % on the control day [27].

### Evaluation of nasal response

Rhinitis was measured using three tests: Linder’s symptoms score scale, changes in nasal cavity volume, and anterior rhinoscopy. SIC with persulphate was considered positive for rhinitis if ≥2 tests were positive and the participant had <2 positive tests on the control day.

#### Linder’s symptoms score scale

Subjective symptoms of rhinoconjunctivitis were scored according to Linder’s symptoms score scale [28, 29]. Participants rated sneezing, rhinorrhea, and nasal congestion from 0 to 3. Ocular symptoms scored 1 point, and itchiness of the nose, ears or palate scored 1 point for each location with itch. An increase of ≥3 points from baseline was considered a positive result.

#### Changes in nasal cavity volume

Swelling of the nasal mucosa was assessed by means of acoustic rhinometry using a Rhinoscan^®^ SRE 2000 (RhinoMetrics A/S, Lynge, Denmark) as previously described [30]. Participant had acclimatized for 20 min before baseline measurements were performed. Total nasal volume (TNV) was measured at 2–6 cm from the nares. A ≥25 % fall in TNV after exposure was considered a positive result [28].

#### Scoring by anterior rhinoscopy

Anterior rhinoscopy was performed and rhinorrhea and nasal congestion were scored separately according to the method proposed by Hytonen [31]. A change in nasal status score of ≥4 points between baseline and exposure was considered a positive response [31].

### Statistical analysis

Data were analyzed using SPSS 22.0 for Windows (SPSS Inc., Chicago, IL, USA). Results for categorical variables are presented as numbers and frequencies, and are compared by the Fischer’s exact test. *P* values ≤0.05 were considered statistically significant (two-tailed tests). Continuous variables were compared with the Mann–Whitney U test and expressed as means ± SDs.

## Results

### Hairdressers

A total of 20 hairdressers were considered eligible for inclusion; one was excluded because of unstable asthma. All were female and the mean age was 31 years (Table 1). Six hairdressers were atopic and three had atopic dermatitis. FeNo was elevated in three, FEV1/FVC was reduced in three, and five showed bronchial hyperresponsiveness in the methacholine challenge. Seven hairdressers used asthma medication and six used rhinoconjunctivitis medication (Table 1). When asked about work-related symptoms, one hairdressers reported asthmatic symptoms (≥2 of the following: wheeze, cough, shortness of breath or hoarseness), one reported rhinitis symptoms (≥1 of the following: nasal itching, runny nose, blocked nose, itchy and watery eyes), and 17 reported both asthmatic and rhinitis symptoms. All 19 hairdressers reported symptoms in relation to hair bleaching and 11 (58 %) admitted that their symptoms could also be provoked by other hairdressing products such as hair dyes, hairsprays, permanent wave solutions, and perfume (Table 1).

**Table 1:** Main characteristics of participants

	Hairdressers (n = 19)	Symptomatic controls (n = 12)	Healthy controls (n = 40)	*P* value^α^
Mean age, years (SD)	31 (10.5)	21 (2.6)	35 (12.9)	0.002
BMI, mean (SD)	22.5 (3.8)	22.4 (3.4)	24.7 (4.3)	1.000
Sex (% female)	19 (100)	7 (58)	43	0.02
Smoking status, n (%)				
Smoker	7 (37)	5 (42)	–	0.79
Never smoker	12 (63)	7 (58)	–	
Atopic dermatitis, n (%)	3 (16)	6 (50)	0 (0)	0.06
Total IgE, mean (SD)	58.3 (76)	156.5 (202)		0.22
Atopy^a^ (%)	6 (32)	10 (83)		0.009
FeNO ≥ 25 ppb, n (%)	3 (16)	5 (42)		0.20
FeNO ≥ 50 ppb, n (%)	1 (5.2)	3 (25)		
Lung function, mean (SD)				
% FEV1	101.7 (9.7)	106.6 (14.8)		0.48
% FVC	105.3 (8.7)	116.8 (14.1)		0.025
FEV1/FVC	84.8 (7.6)	78.9 (6.6)		0.43
Methacholine test				
BHR, n (%)	5 (26)	7 (58)		0.13
Asthma medication, n (%)				
None	12 (63)	0 (0)		
SABA	3 (16)	7 (58)		
SABA + low dose ICS	1 (5)	3 (25)		
SABA + medium dose ICS	1 (5)	2 (17)		
SABA + LABA/ICS	1 (5)	0 (0)		
SABA + LTRA	1 (5)	0 (0)		
Rhinitis medication, n (%)				
None	13 (68)	2 (17)		
OA	2 (11)	8 (67)		
INS	1 (5)	1 (8)		
OA + INS	2 (11)	1 (8)		
OA + antihistamine eye drops	1 (5)	0 (0)		
Work-related symptoms, n (%)				
Rhinitis symptoms	1 (5)	–		
Asthma symptoms	1 (5)	–		
Both	17 (90)	–		
Trigger of symptoms, n (%)				
Bleaching products	19 (100)	–		
Hair dye	9 (49)	–		
Hair spray	4 (21)	–		
Permanent solution	3 (16)	–		
Perfume	3 (16)	–		
Positive stop/resume test, n (%)	16 (84)	–		

*SD* standard deviation, *BMI* body mass index, *SABA* short-acting beta_2_-agonists, *LABA* long-acting beta_2_-agonists, *ICS* inhaled corticosteroids, *LTRA* leukotriene receptor antagonists, *INS* intra-nasal steroid

^a^Defined as 1 ≥positive SPT or 1 ≥positive specific IgE to common inhalant allergens

^α^Comparing hairdressers with controls

### Symptomatic controls

A total of 14 symptomatic controls were eligible for inclusion in the study; two had to be excluded due to unstable asthma leaving ten with concomitant allergic asthma and rhinitis and 2 with non-allergic asthma. The mean age was 21 years and 58 % were female (Table 1). Half had atopic dermatitis. Elevated FeNO was detected in 42 %, FEV1/FVC was reduced in three, and the methacholine challenge was positive in seven. All used asthma medication, whilst only the ten with concomitant allergic rhinitis used rhinitis medication (Table 1).

### Healthy controls

A total of 40 healthy participants had SPT and HRT with persulphates performed.

### Results of SIC

None of the participants reacted to placebo. None of the symptomatic controls developed a nasal or bronchial response when exposed to potassium persulphate in SIC. A total of six (32 %) hairdressers showed a positive reaction to SIC with persulphate; four had a nasal response, and two had a combined bronchial and nasal response (Table 2).

**Table 2:** Characteristics of hairdressers with a positive specific inhalation challenge

ID	Age (y)	WRAS	WRRS	Stop/resume test	Duration of exposure before symptoms (y)	Time from exposure to symptom	Time since last exposure to persulphates (y)	Baseline FEV1/FVC (% of pred.)	MCh PD20 (µg)
2	21	+	+	P	5	Within hours	CE	87 (103 %)	330
5	29	+	+	P	11	Not definable	CE	86.7 (104 %)	N
8	32	+	+	P	1.5	Within minutes	8	85.5 (103 %)	346
10	22	+	+	P	4–5	Within hours	CE	80.6 (96 %)	N
16	23	+	+	P	0.5–1	Within minutes	2	85.4 (101 %) Rever: 12 %	N
19	23	+	+	P	0.5–1	Within minutes	1/3	94.9 (113 %)	N

ID	Age (y)	FeNO (ppb)	T-IgE (kU/L)	Atopy^a^	HRT PP (mg/mL)	HRT SP (mg/mL)	HRT AP (mg/mL)	SIC response	Classification of SIC response
2	21	6.3	73.2	No	N	N	2.5	R A	4 and 8 after 3rd exposure (late reaction)
5	29	6.2	8.7	Yes	N	10.0	10.0	R	1 h after 3rd exposure (immediate reaction)
8	32	*31.2*	46.4	Yes	N	N	5.0	R	1 h after 3rd exposure (immediate reaction)
10	22	7.4	26.6	No	N	N	10.0	R A	1 and 3 h after 3rd exposure (immediate reaction/late reaction)
16	23	13.0	39.8	Yes	10.0	2.5	1.25	R	After 2nd exposure (immediate reaction)
19	23	16.9	63.6	Yes	0.63	–	0.63	R	After 3rd exposure (immediate reaction)

*y* years, *WRAS* work-related asthma symptoms, *WRRS* work-related rhinitis symptoms, *MCh* methacholine challenge, *FeNO* fractional exhaled nitrogen oxide (increased values in italics), *T-IgE* total immunoglobulin E, *HRT* histamine release test, *PP* potassium persulphate, *AP* ammonium persulphate, *SP* sodium persulphate, *SIC* specific inhalation challenge, *N* negative, *P* positive, *CE* currently exposed, *R* rhinitis, *A* asthma

^a^Defined as ≥1 positive SPT to common inhalant allergens

All hairdressers with a positive SIC, reported a positive stop/resume test, whereby their symptoms subsided in periods away from work and deteriorated again when returning to work. They had all been exposed to hairdressing for ≥6 months before developing work-related respiratory symptoms. The typical time interval between initiating work with bleaching products and the appearance of symptoms, was minutes (n = 3), hours (n = 2), or it could not be defined (n = 1). Half of the hairdressers had discontinued their work, and hence were no longer exposed to persulphates on a daily basis. The nasal responses to SIC began within minutes (n = 2), after 1 h (n = 3), and after 3 h (n = 1). The two hairdressers reacting with bronchoconstriction did so after 3 h and 8 h, respectively. The characteristics of hairdressers with negative SICs are presented in Table 3.

**Table 3:** Characteristics of hairdressers with a negative specific inhalation challenge

ID	Age (y)	WRAS	WRRS	Stop/resume test	Duration of exposure before symptoms (y)	Time from exposure to symptoms	Time since last exposure to persulphates (y)	Baseline FEV1/FVC (% of pred.)
1	52	+	+	N	3	Within hours	5	86 (109 %)
3	45	+	+	P	20	Within hours	CE	83 (104 %)
4	49	+	+	P	28	Within minutes	CE	65 (81 %) Rever: 2.9 %
6	30	–	+	P	10	Within minutes	3	81 (97 %)
7	27	+	+	P	1	Not definable	1	84.9 (103.3 %)
9	20	+	+	P	1	Within minutes	CE	98 (116 %)
11	43	+	+	P	20	Within hours	CE	83 (103 %)
12	23	+	+	N	3	Within hours	CE	74 (88 %) Rever: 12 %
13	46	+	–	N	2	Not definable	3	83 (103 %)
14	27	+	+	P	7	Within minutes	CE	83 (99 %)
15	31	+	+	P	4	Within minutes	1/2	75 (90 %) Rever: 7.8 %
17	20	+	+	P	4	Not definable	1/6	91 (108 %)
18	29	+	+	P	8	Within hours	CE	91 (109 %)

ID	Age (y)	MCh PD20 (µg)	FeNO (ppb)	T-IgE (kU/L)	Atopy^a^	HRT PP (mg/mL)	HRT SP (mg/mL)	HRT AP (mg/mL)
1	52	N	*25*	3.6	No	1.25	0.63	1.25
3	45	N	9.9	6.7	No	N	5.0	2.5
4	49	N	16.0	2.2	No	10.0	10.0	5.0
6	30	400	*64.1*	155	No	N	10.0	5.0
7	27	N	19.3	127	D	N	5.0	2.5
9	20	N	6.6	<2	No	N	10.0	5.0
11	43	N	7.1	22.5	No	N	10.0	5.0
12	23	N	21.8	122	No	10.0	5.0	2.5
13	46	626	12.1	35.4	No	N	5.0	1.25
14	27	N	11.8	3.7	No	N	10.0	5.0
15	31	720	9.0	308	D	5.0	2.5	1.25
17	20	N	9.0	4.3	No	10.0	2.5	2.5
18	29	N	19.7	58.3	No	N	N	N

*y* years, *WRAS* work-related asthma symptoms, *WRRS* work-related rhinitis symptoms, *MCh* methacholine challenge, *FeNO* fractional exhaled nitrogen oxide (increased values in italics), *T-IgE* total immunoglobulin E, *HRT* histamine release test, *PP* potassium persulphate, *AP* ammonium persulphate, *SP* sodium persulphate, *SIC* specific inhalation challenge, *N* negative, *P* positive, *CE* currently exposed, *D* dermographism, *R* rhinitis, *A* asthma

^a^Defined as ≥1 positive SPT to common inhalant allergens

### Quantification of potassium persulphate

Before exposure, the amount of particles sized 0.1–10 µm inside the provocation chamber ranged from 7 to 18 µg/m^3^ and the number of ultra-fine particles was 347–1260/cm^3^. No additional ultrafine particles were detected during a 3 × 5 min exposure to 50 g pure potassium persulphate in the Erlenmeyer flask.

The mean amount of particles sized 0.1–10 µm measured during a 5-min exposure to a mixture of 30 g potassium persulphate and 20 g lactose ranged from 0.25–0.57 mg/m^3^ and the proportion of potassium persulphate to lactose powder in the flask was 3:2. Thus, the estimated concentration of potassium persulphate in the air during a 5-min exposure was 150–340 µg/m^3^ with a mean of 240 µg/m^3^ and a standard deviation of 0.6 µg/m^3^. During the 20-min pause in between exposures, the amount of particles in the air returned to baseline values.

### SPT results

In two hairdressers, the negative control was positive due to dermographism and therefore their SPTs could not be evaluated (Table 4). All participants reacted to the positive control (histamine), whilst none were positive to latex, chlorhexidine, or any of the three tested persulphates (Table 4).

**Table 4:** Results from skin prick tests

	Hairdressers (n = 19)	Symptomatic controls (n = 12)	Healthy controls (n = 40)
Positive control (p/n)	19/0	12/0	40/0
Negative control (p/n)	2/17	0/12	0/40
Potassium persulphate (p/n) Conc. (%)	0/17	0/12	0/40
2	–	–	–
5	–	–	–
7.5	–	–	–
Ammonium persulphate (p/n) Conc. (%)	0/17	0/12	0/40
2	–	–	–
5	–	–	–
7.5	–	–	–
10	–	–	–
20	–	–	–
Sodium persulphate (p/n) Conc. (%)	0/17	0/12	0/40
2	–	–	–
5	–	–	–
7.5	–	–	–
10	–	–	–
20	–	–	–

*p* positive, *n* negative

### Results of HRT with persulphates

Of the six hairdressers with a positive SIC, four (66.7 %) did not react to HRT with potassium persulphate or sodium persulphate at any of the tested concentrations. In contrast, all six hairdressers with positive SICs released histamine in response to ammonium persulphate at concentrations ranging from 0.063 to 1 %. So did also 96.2 % of symptomatic controls and healthy controls. For all three persulphates, the lowest concentration producing histamine release in the controls and healthy controls was 0.125 %, whilst some of the hairdressers reacted to concentrations of 0.06 %. None of the participants showed histamine release to any of the persulphates in concentration of 0.031 %.

## Discussion

### SIC

In this study, we aimed at improving the currently validated SIC with persulphate. The improvements consisted of: a more rapid approach; using the “stirring method” instead of the “tipping method”; and assessing not only asthma but also rhinitis.

When Munoz et al. validated the realistic method [20], repeated exposures on consecutive days were performed with a mixture of potassium persulphate and 150 g lactose using the tipping method. The duration of the exposure was 10 min each day, and the dose of potassium persulphate was increased from 5 to 30 g over 4 days until a positive reaction occurred. The patient was hospitalized during the entire procedure. The method proved safe and a sensitivity of 100 % and a specificity of 87.5 % for diagnosing occupational asthma were reported.

In our method, we skipped the first 3 days with low exposure, and went straight to exposing the patient to 30 g of potassium persulphate. Instead of 10 min exposure we performed 15 min exposure. To reduce the risk of adverse reaction, exposure was performed step-wise; 5 min at a time with 20 min pauses in between, and severe asthmatic reactions and/or anaphylactic reactions to bleaching products were considered exclusion criteria.

Given that none of the symptomatic controls with allergic asthma and rhinitis reacted to SIC, it seems that the proposed method has a high specificity for persulphate-induced asthma and rhinitis. In this group of hairdressers, SIC produced a nasal response in 33 % (6/18 with work-related rhinitis symptoms) and a bronchial response in 11 % (2/18 with work-related asthma symptoms).

We registered no adverse events or severe asthmatic reactions although our exposure was higher than Munoz’ on the fourth day. Hence, it seems that the rapid method is safe when tested in patients without a history of severe asthmatic reactions or anaphylactic reactions to bleaching products.

We have several reasons for using the level of exposure we did. Firstly, we chose 3 × 5 min exposure to better mimic the hairdressers’ exposure during a typical working day. Since hairdressers are mainly exposed to persulphates when they mix bleaching powder with hydrogen peroxide [32], we wanted to mimic this process. We estimated that a typical hairdressers performs this process three times a day. Secondly, the ratio of persulphate to lactose powder was changed as to better mimic the level hairdressers are exposed to in their daily practice. During mixing of the paste that is applied to the clients hair, 20–80 g bleaching powder [33], containing up to 60 % persulphate (12–48 g) [1], is typically used. We therefore used a ratio of persulphate to lactose powder of 3:2 (30 g persulphate:20 g lactose powder). To obtain a more uniform and reproducible exposure, we used a magnetic stirrer. In our study, the participants were exposed to levels of up to 0.34 mg/m^3^ for 3 × 5 min during SIC. The permissible threshold limit value of exposure to potassium persulphate, as defined by the Occupational Safety and Health Administration in the United States, is a time weighted average (TWA) of 0.1 mg/m^3^ during a typical working day of 8 h. According to the excursion limit of potassium persulphate, the TWA should not be exceeded more than 3 times for no longer than 30 min during a working day. Hence, the TWA was exceeded during our exposure, but the excursion limit was respected.

A limitation of our approach is that the patients were sent home after 8 h of observation in the clinic. This is convenient for the patient, but it introduces a potential bias. If the patient develops a positive nasal or bronchial response during this period at home, it is difficult to interpret whether the response was caused by exposure to persulphates or by exposure to other allergens encountered outside the hospital. However, in our study, all hairdressers reacted whilst being monitored in our department, so it is unlikely that this is a problem in our results.

Another limitation of our study is that the included hairdressers were merely under suspicion of having occupational asthma and rhinitis, but they were not clear-cut cases, which explains why only some hairdressers had a positive reaction to SIC. Firstly, they did not have serial peak flow measurement at and away from work performed prior to inclusion. If we had included only patient with a peak flow pattern suggestive of occupational asthma it might have improved the sensitivity of the test for detecting persulphate-induced asthma. Secondly, many had normal findings in spirometry, FeNO, and the methacholine challenge suggesting that they did not in fact have asthma although they reported asthmatic symptoms. Third, although persulphates are considered the major cause of occupational asthma and rhinitis in hairdressers [34] more than half reported that their work-related respiratory symptoms could also be provoked by other hairdressing products suggesting that their respiratory symptoms were not merely caused by persulphates. Also, some of the hairdressers had not been active hairdressers for several years and therefore were not still exposed to persulphates meaning that they could have lost airway responsiveness. Taken together, several factors exist that could explain why not all hairdressers reacted to the SIC and consequently the sensitivity of our approach cannot be determined.

### HRT and SPTs with persulphate salts

This is the first study, to the best of our knowledge, to report results of HRT with persulphates. We found that persulphates, especially ammonium persulphate, induced non-IgE-mediated histamine release in both hairdressers and controls. Additionally, most of the SIC-positive hairdressers did not show histamine release. Ammonium persulphate has recently shown to have oxidative activity capable of promoting degranulation of human mast cells and basophils [13]. Thus, persulphates stimulate nonspecific non-IgE-mediated histamine release even in individuals without symptoms of persulphate-induced respiratory diseases, voiding the use of HRT to document asthma or rhinitis caused by persulphates.

We performed SPTs in duplicate with all three persulphates simultaneously, at concentrations as high as 20 %. To our knowledge, this has not been done before. We did not register any positive SPTs with persulphates in any of the participants, although all responded positively to the histamine control. Given the high persulphate concentrations applied, lack of positive reactions does not seem to be caused by using excessively low dosage. In addition, by testing all three persulphates, we ensured that we would not miss any patient sensitized to only one of the three persulphates [35].

Although several reports of positive SPTs with persulphates exist [8, 19, 21, 23], an equal amount of studies have failed to produce positive reactions [7, 14, 30, 31]. Moreover, in some patients, positive reactions are not reproducible over time [36].

The fact that specific IgEs to persulphates have been detected in only three [10, 37], out of more than 40 reported positive SPT cases, indicates that positive SPT reactions are caused by nonspecific non-IgE mediated histamine release. Indeed, when researchers with a method capable of detecting specific IgE to persulphates tested five patients with positive SPT reactions, they found that only two had demonstrable specific IgE [10], suggesting that the remaining positive SPT reactions were not mediated by IgE.

All in all, the majority of positive SPT reactions appear to be caused by direct histamine release rather than IgE-mediated mechanisms. Moreover, they have been reported by only a fraction of investigators, and are not always reproducible. Taken together, this indicates that SPTs cannot be applied to testing for persulphate-induced asthma and rhinitis.

## Conclusions

The new rapid SIC with potassium persulphate proved safe when tested in hairdressers without a history of previous serious asthmatic reactions and had a high specificity for diagnosing persulphate-induced asthma and rhinitis. Based on our results, neither histamine release nor SPTs with persulphates appear adequate in predicting asthma and rhinitis caused by persulphates.

## Abbreviations

ACS: American Chemical Society
BHR: bronchial hyperresponsiveness
CAS: Chemical Abstract Service
FeNO: fractional exhaled nitric oxide
FEV_1_: forced expiratory volume in 1 s
HRT: histamine release test
IgE: immunoglobulin E
rpm: revolutions per minute
SIC: specific inhalation challenge
SPT: skin prick test
TNV: total nasal volume
